# Analysis of Information Availability for Seismic and Volcanic Monitoring Systems: A Review

**DOI:** 10.3390/s22145186

**Published:** 2022-07-11

**Authors:** Santiago Arrais, Luis Urquiza-Aguiar, Carolina Tripp-Barba

**Affiliations:** 1 Departamento de Informática y Ciencias de la Computación (DICC), Escuela Politécnica Nacional, Quito 170517, Ecuador; santiago.arrais@epn.edu.ec; 2 Instituto Geofísico (IG), Escuela Politécnica Nacional, Quito 170517, Ecuador; 3 Departamento de Electrónica, Telecomunicaciones y Redes de Información (DETRI), Escuela Politécnica Nacional, Quito 170517, Ecuador; 4 Faculty of Informatics Mazatlan, Autonomous University of Sinaloa, Mazatlán 82117, Mexico; ctripp@uas.edu.mx

**Keywords:** information security, availability, seismic–volcanic monitoring, seismic–volcanic networks, seismological data centers, algorithms

## Abstract

Organizations responsible for seismic and volcanic monitoring worldwide mainly gather information from instrumental networks composed of specialized sensors, data-loggers, and transmission equipment. This information must be available in seismological data centers to improve early warning diffusion. Furthermore, this information is necessary for research purposes to improve the understanding of the phenomena. However, the acquisition data systems could have some information gaps due to unstable connections with instrumental networks and repeater nodes or exceeded waiting times in data acquisition processes. In this work, we performed a systematic review around information availability issues and solutions in data acquisition systems, instrumental networks, and their interplay with transmission media for seismic and volcanic monitoring. Based on the SLR methodology proposed by Kitchenham, B., a search string strategy was considered where 1938 articles were found until December 2021. Subsequently, through selection processes, 282 articles were obtained and 51 relevant articles were extracted using filters based on the content of articles mainly referring to seismic–volcanic data acquisition, data formats, monitoring networks, and early warnings. As a result, we identified two independent partial solutions that could complement each other. One focused on extracting information in the acquisition systems corresponding to continuous data generated by the monitoring points through the development of mechanisms for identifying sequential files. The other solution focused on the detection and assessment of the alternative transmission media capabilities available in the seismic–volcanic monitoring network. Moreover, we point out the advantage of a unified solution by identifying data files/plots corresponding to information gaps. These could be recovered through alternate/backup transmission channels to the monitoring points to improve the availability of the information that contributes to real-time access to information from seismic–volcanic monitoring networks, which speeds up data recovery processes.

## 1. Introduction

Commonly, seismological data centers (SDC) around the world are continually adding new technologies for data acquisition and processing, data storage, information analysis, and information diffusion. So, SDCs have reached their current monitoring networks by increasing instrumental networks and repeater nodes of different capabilities at different stages. Instrumental networks of SDCs consist of various sensor types such as seismic sensors, accelerometers, inclinometers, high-precision GPS, monitoring cameras, pressure sensors, infrasound sensors, gas measurement sensors, and others [1,2,3]. These specialized sensors require other components such as data acquisition systems, data loggers, transmission media, energy sources, and physical infrastructure. Once new sensing data are generated, they are sent in continuous mode or time intervals, mainly using TCP/IP protocol stack through a data transmission media assigned to each terminal station.

Communication media used commonly in SDCs are microwave, satellite, Wi-Fi, optical fiber, 3G/4G network, and the Internet [4,5]. Some network topologies combine communication media by using repeaters nodes until reaching SDC. These centers host the acquisition, processing, and storage systems for real-time monitoring, analysis, diffusion, and other processes [6].

Sometimes, data transfer is discontinuous because of internal or external factors. Among the latter case, radio frequency interference and weather conditions are prevalent in wireless links. On the other hand, internal factors are related to the transmission medium’s technological limitations, power backup failures, problems associated with the retrieval of information from the acquisition systems, and underused alternative channels in the repeater nodes. For example, IG-EPN (Instituto Geofísico, Escuela Politécnica Nacional, Ecuador) located in South America has annually identified that the overall performance of data transmission networks is not at the best level (less than 90 percent) [7,8], due to the factors mentioned above and others, such as the diversity of data acquisition systems and manual procedures for the data recovery process used in some SDCs.

**Figure 1:**
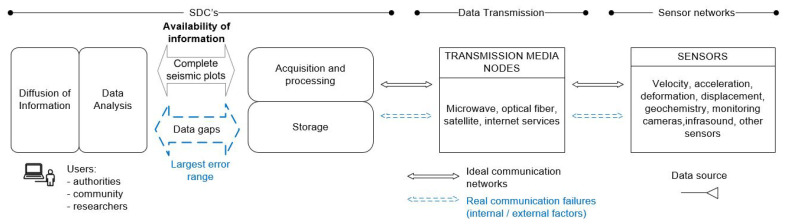
Seismic-volcanic monitoring systems. A general diagram.

Figure 1, “seismic–volcanic monitoring systems: a general diagram”, aims to visualize the involved processes from the source to the users. Where the problem representation contrasts an ideal system in front of a real system, processes could be improved with the contribution of this research This study aims to identify the leading causes that could increase the error range in data acquisition, analysis, and diffusion of information, as well as identify some actions that have been taken to solve these problems on specific seismic–volcanic networks. Currently, SDCs have to process this information with possible accuracy failures or delays in processing time due to data gaps.

Information security focuses on the assurance of data systems through the compliance of processes, standards, and mechanisms that improve the confidentiality, integrity, and availability of information, regardless of where it is located. Regarding information availability, operational continuity focuses on information reliability at the source, storage, processing, transmission, or access to information [9]. Operational continuity also includes evaluating risks and threats, establishing action plans, applying standards, implementing good practices that improve the management of information assets, and guaranteeing service continuity [10].

From the information security perspective [11,12,13], confidentiality is defined as the characteristic that allows access to information only to authorized users. Integrity refers to ownership to protect data from alterations or modifications unauthorized, and availability considers the timely delivery of data when the authorized user requires it. The information availability requires that the service must be ready for the user (people, processes, and applications) [14] when needed and with authorization; therefore, the information system components must be in operation and avoid service interruptions, hardware failures, communications, and power outages [12]. Nonetheless, availability from an information security perspective considers the loss and/or acceptable data interruption in a specified period. High availability and redundancy systems can detect failures and minimize such disruption [15], among others.

Organizations responsible for seismic and/or volcanic studies worldwide (SDCs) have “seismic data” as their primary information asset to determine early warnings [2,16]. These data are generated by the instrumental monitoring networks and carried over different transmission media toward the data centers. Subsequently, data are received in acquisition and processing systems. Then, they are stored for use in the information interpretation and diffusion processes, as well as future phenomenon research. For example, in these studies [17,18,19], even though organizations are different and have seismic monitoring responsibilities or volcanic monitoring, they have similarities in their structure for information processing.

At communication systems for seismic and volcanic monitoring, the most compromised component of the information security triad is availability. Therefore, this study is motivated by the availability vulnerabilities, the possible risks of communication outages that affect data quality, its incidence on early warnings, and response times. The goal is to propose an information security strategy with mechanisms for increasing the availability of information in instrumental networks and their interaction with acquisition and processing systems inside SDCs. Therefore, the study does not focus on risk management and possible seismic–volcanic impacts on the population, impacts on human lives, and risks for the population.

The diversity of worldwide seismic and volcanic monitoring networks has been considered in this review, due to seismic and/or volcanic monitoring centers being structured in blocks or similar processes that include: measurement of seismic–volcanic activity, data transmission, acquisition, processing, analysis, interpretation, and diffusion of information.

The literature review in this research will summarize the most important methods and solutions that contribute to the availability of information in data acquisition systems and data transmission networks for seismic and volcanic monitoring. In this way, this work relates to recent studies, mainly since 2015, on the most recommended protocols and algorithms for recognizing factors that affect the real-time transmission, data recovery processes, and the information from instrumental networks designed for geological phenomena monitoring. This article review criterion is due to software updates for acquisition systems and features of current models of sensors, digitizers, and transmission media used in seismic–volcanic monitoring networks.

Section 2 introduces the methodology, including research questions, search strategies, study selection, study quality assessment, data collection, and data analysis to achieve a summary of relevant studies. Section 3 reviews seismic studies and results about instrumental networks, data acquisition and processing, seismic data standards, and early warning trends. Section 4 provides an analysis of the proposed research questions. Finally, Section 5 concludes the research findings in this literature review, the threats to data acquisition systems and seismic monitoring networks, and possible future solutions.

## 2. Methodology Validation

To know the advances in a topic and identify gaps in the research, there is the methodology known as a systematic literature review (SLR) which is used as a predefined research strategy for performing research methodology [20,21]. The main objective is to provide an overview of the research area and identify the amount and type of research and the available results. It is also essential to map published frequencies over time to understand trends and identify forums where research in the area has been presented [22]. As a result, the followed review procedure is composed of six stages: (1) research questions, (2) search strategy, (3) study selection, (4) study quality assessment, (5) data collection, and (6) data analysis.

### 2.1. Research Questions

A total of three research questions were designed for the review. These questions will allow delving into specific aspects of seismic monitoring networks from the information availability approach. For each question, a purpose and criteria have been described in this study to find relevant information as shown below:

**RQ1**  *What are the mechanisms used in instrumental monitoring networks that contribute to availability of information?* The aim is to identify protocols, algorithms, and techniques focused on real-time data transmission, as well as to recognize factors that affect the availability of information.
**RQ2**  *What are the regulations and standards proposed by seismological organizations about the availability of information for data acquisition and processing systems?* The goal is to identify operational conditions and requirements for data acquisition systems in data centers.
**RQ3**  *What are seismological network trends in IT infrastructure that improve the information recovery from instrumental networks?* We seek to recognize trends in connectivity between seismological data centers and their monitoring networks.

### 2.2. Search Strategy

The search strategy composes of a search pattern, which describes the repositories used in this review. In addition, we considered three viewpoints for research questions analysis: population, intervention, and outcome. Finally, the search strings were built using Boolean ANDs and ORs, in a defined time range.

The *Search Pattern* used combinations of key terms in digital libraries such as Scopus, Science Direct, ACM Digital Library, and IEEE Xplore. Journals or conference proceedings have been included to select the main studies of this work.

The *Population* defined for this study are: seismological data centers, seismic–volcanic monitoring network organizations, and the scientific community. These groups are focused on the diffusion of early warnings of seismic and volcanic events.

The *Outcome* has been defined through two main factors, which could support the availability of information in the SDCs. The first factor refers to designing a mechanism to retrieve seismic data, and the second factor considers a validation method to add redundant networks.

Procedures considered in *Intervention* help to identify algorithms applied in data acquisition and seismic transmission networks. Likewise, it includes seismic data formats, protocols, and software solutions for data storage and processing.

It is essential to mention that a recommended way to create the search string is to structure them in terms of population, intervention, comparison, and outcome [21]. The selected search period is from the years 2000 to 2021 because, since 2000, most SDCs had already migrated their analog to digital systems, including transmission media and systems for the acquisition and processing of seismic data. The expressions were constructed using logical operators for searches (AND and OR), shown in Table 1.

The most important terms were “data acquisition and processing systems“ for seismic–volcanic monitoring phenomena, “algorithms and protocols“ related to the availability of information, “seismic networks“ description, and “seismic data standards“ for data transmission. The search results allow the selection of publications related to this study.

### 2.3. Study Selection

This section is built based on the structure of a systematic review (Kitchenham guide—Section 5. Conducting the review) [20,21]. From this approach, two criteria identify relevant studies (51 articles) and potentially relevant studies (29 articles), depending on how much they directly contribute to the research questions. Moreover, the third criterion refers to non-relevant studies (130 articles), i.e., those that do not respond to the RQs raised. In addition, duplicate studies were also filtered. This study reviewed inclusion and exclusion criteria, which are essential features to determine the literature review’s relevance. The exclusion criteria were:

- Studies that do not relate to the proposed research objectives;
- Studies that delve into seismic risk management;
- Specific geological studies;
- Studies without peer review.

The inclusion criteria are listed below:

- Publications in English and Spanish;
- Publications since 2000;
- Publications type: articles (journals, conferences, surveys, SLR), reports, book sections;
- Relevant publications related to:
  1. Security of information;
  2. Availability;
  3. Data acquisition;
  4. Algorithms;
  5. Routing protocols;
  6. Seismic networks;
  7. Acquisition and processing systems;
  8. Data centers;
  9. Seismic monitoring;
  10. Seismic stations;
  11. Components and infrastructure.
- Official websites, reports of seismological organizations.

A summary of results found with the searching string (outcome, inclusion, and intervention) and selection criteria mentioned above is presented in Table 2. The largest number of articles has been found with the proposed search strings for RQ1 with 1003 articles. Besides RQ2 and RQ3, there are 601 and 334, respectively. On the other hand, combined relevant terms that retrieve more related articles in the string are: seismic data, acquisition, processing, algorithms, and protocols. Concerning databases, Science Direct has more quantity of related articles for this study (39%) in comparison to Scopus (24%), ACM (20%), and IEEE Xplore (17%). The goal of this stage has been to obtain relevant studies, making it necessary to apply a selection based on the inclusion criteria and study quality assessment mainly.

With the search results, a more detailed review was made of the potential studies. A first step is to detect duplicate works in our preliminary search because many works are indexed in several repositories. After that, we made classifications based on their relevance to our review according to the following criteria:

- *Not relevant studies.* Studies that do not contribute to answering research questions RQ1, RQ2, or RQ3.
- *Potential relevant studies.* Within this articles group, we identified an indirect relationship between the objective mentioned in RQ1 and the solutions proposed for data transmission assurance in real time. However, these articles do not point to seismic data processing or seismic data acquisition. These do not refer to practical or experimental cases for EEW either. As an example, we can mention WSN mobile solutions, as well as LoRaWan applications for networks in urban areas with a high demand for data traffic.
- *Relevant studies.* In this group, we selected 51 highly relevant works because these identified proposals and solutions directly related to the seismic–volcanic monitoring networks, as well as improvements and alternatives to optimize the seismic data acquisition and processing systems. On the other hand, we also identified ad hoc point solutions for seismic–volcanic monitoring networks. It is important to recognize that some studies do not consider the approach proposed in this research. The scope of these studies is partial. In other cases, the proposed solutions cannot be applied to different seismic network environments.

The documents were analyzed, compared, and used as referents to identify knowledge gaps in the security of information-oriented data acquisition systems and data transmission networks for seismic–volcanic monitoring.

**Figure 2:**
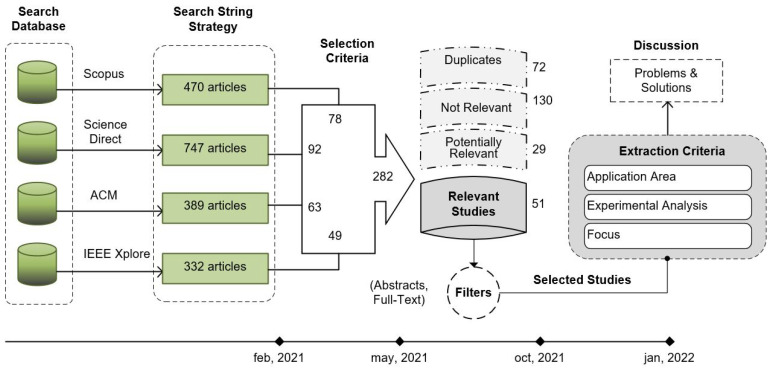
Research review process.

Figure 2 shows each of the stages in the review process designed for this study. It starts in the database search, passing by the string strategy that offers 1938 articles until December 2021. After the selection criteria (inclusion and exclusion), 282 articles were selected and reduced to 51 when only relevant studies are extracted. Finally, filters based on the content of papers were applied to selected articles. To identify the possible research gaps that fit the objectives of this review, we addressed four groups: (a) articles oriented to seismic data transmission networks, (b) seismic data acquisition and processing systems, (c) seismic data standards, and (d) early warning trends.

### 2.4. Study Quality Assessment

Based on Kitchenham guidelines for quantitative studies [21], we evaluated quality aspects in the publication content of the 51 selected studies. The quality assessment of the selected works is summarized in Table 3. The general questions that we use for this purpose are:

- Q1: Are the aims clearly stated?
- Q2: Is there a sampling strategy?
- Q3: Is the sample representative of the population to which the results will generalize?
- Q4: Is there a comparison or control group?
- Q5: Is the application area clearly defined?
- Q6: Are the data collection methods adequately described?
- Q7: Were the basic data adequately described?
- Q8: Is the purpose of the analysis clear?
- Q9: Are all study questions answered?
- Q10: Are important effects overlooked?

**Figure 3:**
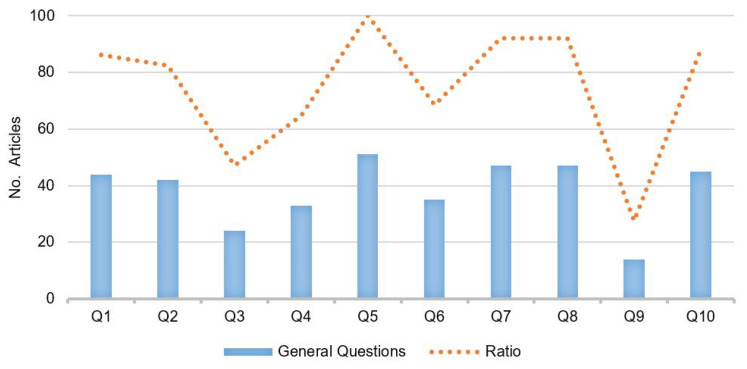
Study quality assessment.

With the relevant studies and groups identified, the quality assessment summary in Figure 3 shows that, on average, each article answers 70% of the quality questions applied. More than 45 articles answer five questions, which can partially contribute to possible solutions for the RQs raised above (Stage A). On the other hand, regarding the research question Q5 about defined application areas, 51 studies are directly related to seismic–volcanic monitoring systems, where more than 44 investigations raised clear objectives, analysis, and details of the data used (Q1, Q7, and Q8). In addition, 33 articles show results based on comparative or control groups (Q4).

In contrast, 10% of these overlooked important effects (Q10), and only 14 studies (Q9) propose explicit research questions. Concerning the results, 27 studies propose ad hoc solutions with non-representative samples; therefore, it cannot be generalized in other seismic–volcanic monitoring networks (Q3).

Due to the aforementioned, the relevant information search to resolve research questions raised is not consolidated as a broad solution. Therefore, it is necessary to address the four identified groups, and to establish a proposal that complements the studies and advances that have already been made to date. Finally, this study focussed on the availability of information improvement for seismological data centers.

### 2.5. Data Collection

This step is defined as a process of data extraction based on general information of selected articles where units of analysis proposed, justification of the study, sampling strategy, methodology, publication year, and others are compared. It was focused on the selected primary studies and addressed the proposed research questions.

**Figure 4:**
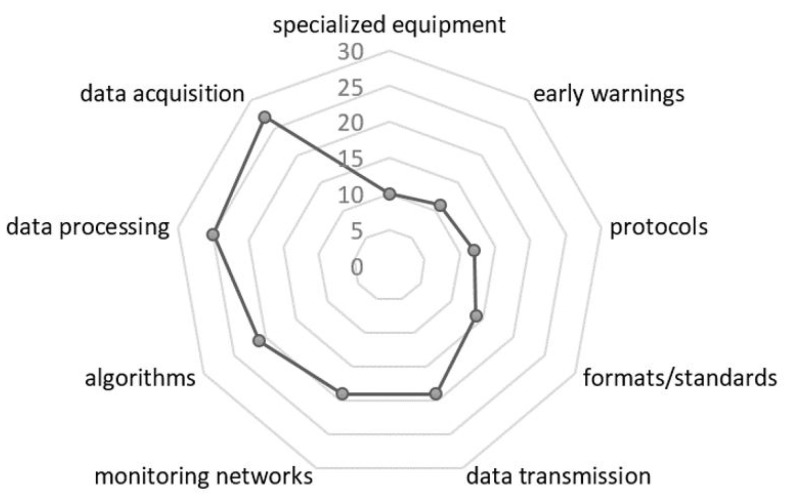
Identified Issues.

Figure 4 represents topics identified within the 51 relevant studies, where researchers have placed greater emphasis on identified problems for at least one of the four identified areas, and solutions have been prioritized for the following topics. Seismic data acquisition (30 articles) is the most researched area, followed by data processing (25 articles), most of which are based on the development of algorithms (23 articles). Furthermore, several authors have emphasized solutions to optimize seismic monitoring networks and ad hoc alternatives for data transmission (19 articles). On the other hand, it can be seen that the number of studies for the development of communication protocols (15 articles) is less because several investigations assume ideal systems without a loss of information; in addition, redundancy for seismic data is not considered. Regarding specialized equipment, there is great progress and diversity among sensors and dataloggers that meet quality standards, robustness, low energy consumption, storage capacity, and compatibility with communication formats and acquisition systems for more than a decade; therefore, this field is currently developed (less than nine articles). Finally, there are specific studies (11 articles) in the EEW field related to exclusively to find different methods for data processing time reduction and generate early warnings with greater precision. However, from the EEW approach, greater emphasis has not been placed on the stages behind the processing, such as data transmission and acquisition, which contribute significantly to the availability of information in the SDCs.

### 2.6. Data Analysis

In this section, 51 articles are identified which contribute to the objectives of this study. Below, an overview includes the research area, justification for the study, the sampling strategy, the methodology, and the publication year. Application areas in recent studies are data transmission networks, data acquisition and processing, seismic data standards, and early warning trends.

- Data transmission networks.

Behr et al. [23] proposed a strategy for a virtual seismologist (VS) through an open-source real-time monitoring software for SC3 to test and evaluate the EEW algorithm. The objective was latency reduction, upgrade software in network components, and reconfiguration in dataloggers. Within this methodology used for VS(SC3) evaluation, Monte Carlo simulation was applied to optimize alert times, P- and S-wave delays, filtering, phase detection, and true and false alert detections. Stubailo et al. [24] made a latency data recognition for seismic data transmission as a crucial parameter in EEW. A partial solution is proposed through the reconfiguration of datalogger parameters, deploying software upgrades in seismic networks. Other studies by Weber et al. [29], Adams et al. [30], and Vidal et al. [28] described seismic sensors, data-logger components, and methods used in specific networks for real-time monitoring.

Scarpato et al. [27] developed ad hoc software applied in wireless data transmission systems, as well as acquisition and visualization systems, for a specific volcanic area.

The main measured parameters were delay, standard deviation, and packet loss statistics. Other QoS metrics such as real time delay, availability, and robustness (fault tolerance) identified network performance.

Zhong et al. [38] proposed an ad hoc development wireless transmission for heterogeneous networks in seismic exploration. A test environment considered acquisition, wireless transmission, and data control. The system used IEEE 802.15.4 and IEEE 802.11b/g. Reddy et al. [37] proposed a procedure and simulation for energy consumption reduction with a large number of hops. Network architecture was based on the IEEE 802.11ad. Evaluation parameters were: average power consumption, end-to-end latency in contrast with operating frequency, bandwidth, transmit power, and receiver sensitivity.

Kaur et al. [32] and Piyare et al. [33] reviewed TDMA protocols and the advantage of using WSN and LoRa networks. The objective was to improve energy consumption, transmission latency, traffic, bandwidth use, and others for seismic monitoring applications. Iqbal et al. [34] made a WSN analysis for seismic data acquisition networks; the study considered data throughput and transmission time from wireless geophones to gateway node in a wireless network architecture based on IEEE802.11af standards.

Mothku et al. [35] proposed a mechanism to improve reliable data transmission in a wireless sensor using Markov decision processes, because of wireless link fluctuations in faulty regions. The model helped to improve the packet level reliability with stringent delivery delay requirements in the presence of faulty nodes. The measured parameter, packet redundancy levels in the network coding process, applied link loss rates and redundancy levels.

Zhou et al. [31] proposed a routing protocol for underwater sensor networks (UWSN), i.e., “Q-learning-based localization-free any path routing (QLFR) protocol” which focused on holding time mechanism for packet forwarding, and analysis of routing protocol performance. The goal was to decrease high energy consumption and large latency in the underwater environment using Q-learning-based localization-free anypath routing.

Li et al. [25] proposed a data compression algorithm to decrease the size of SEG-Y files and the conversion of miniSEED files for the transmission and storage of large amounts of seismic exploration data. The compression algorithm was developed with the Lempel–Ziv–Markov chain algorithm, providing experimental results. Helal et al. [36] proposed a seismic data compression model through the convolutional neural network (CNN). The main goal was to contribute to memory optimization in transmission equipment, and to preserve seismic information for rebuilding.

- Data acquisition and processing.

Dost et al. [39] described the most common seismic data formats; common conversion programs; standards for exchange and data storage; as well as the format structure of SEED, SAC, GSE, CSS, SEISAN, miniSEED, ASCII, ESSTF, and conversion methods. Ringler et al. [26] made a summary of the Standard for Exchange of Earthquake Data (SEED format), as well as the structure and advantages of dataless SEED, which was the most common format used to share metadata.

Abdelwahed et al. [42] developed an ad hoc application for seismic waveform analysis in a specific organization. Cordery et al. [40] proposed a processing workflow to improve the quality of the final processed data. The goal was to significantly decrease noise, and to recover missing signals of seismic broadband sensors. Y. An et al. [51] proposed a workflow for automatic fault recognition in seismic data using deep convolutional neural networks (DCNNs). It required conversion of geological project files to other formats.

Krischer et al. [56] published the ObsPy Python library developed for seismological packages and workflows, through the integration and re-purposing of established legacy codes, using the data processing time, conversion formats, and modern workflows composed in Python. The study was proposed because some seismological tools face several hurdles to generalize into scientific Python system, such as special file formats, unknown terminology, and no suitable replacement for a non-trivial piece of software. Hosseini et al. [46] proposed ObspyDMT Python, a software tool used for the query, retrieval, processing, and management of seismological data sets. It allowed some repetitive and complex diary seismological tasks such as data storage, preprocessing of information, instrument correction, and quality control routines. Other previous studies (e.g., Beyreuther et al. [44] and Megies et al. [45]) also proposed the Python toolbox for seismology and SAC file conversion, unifying a wide variety of computing platforms, file formats, methods for accessing seismological data through information preprocessing standards, as well as libraries to access and process seismological waveform data and metadata.

For data processing, seismic signal deconvolution methods were applied to improve filtering effects or attenuation at the source of seismic waves, and Pilikos et al. [41] proposed a method to reconstruct seismic data using a relevance vector machine (RVM). Experiments were conducted on synthetic and field data sets. Anvari et al. [47] proposed a method to reduce random seismic noise and seismic signal attenuation using Hankel sparse low-rank approximation. Their sampling strategy was used through acquisition parameters to simulate synthetic data composed of 76 traces. A 25 Hz Ricker wavelet generated the seismic section, and the seismic noise was contaminated with white Gaussian random noise. The test results of noise attenuation were compared with the NLM, OptSLR, DRR, and OptWSST methods using land field data and synthetic seismic data.

Wang et al. [48] proposed an automatic picking method for multi-mode surface-wave dispersion curves with unsupervised machine learning to reduce time on human–machine interaction, improve efficiency, and increase accuracy of data processing. Seismic data were changed to 3D dispersion images through GMM clustering, DBSCAN algorithms, and filters for dispersion curves. Results were analyzed on synthetic tests and field data. Zhao et al. [49] developed open-source software for automatic phase detection of seismic waves using a deep learning model. The model was trained with 700,000 waveforms from the Northern California earthquake catalog and showed detection accuracy, identification of events and noise, and low computing resources for processing P- and S-wave arrival times. The designed software includes an application terminal interface, docker container, data visualization, and SSH protocol data transmission, and also supports SAC, MSEED, and NumPy array.

Bin et al. [52], 2021, made a review of IoGN sensing devices, algorithms, architecture, and applications for seismic data acquisition units and data servers. The main techniques that could be applied to IoGN are denoising methods, including compressed sensing (CS) and autoencoders (AE) used to reduce seismic noise. IoGN sensing devices are accelerometers and geophones. Common ADC 16/24-bit resolutions and communication modules use IEEE 802.11 or cellular network standards, as well as ZigBee and GPRS communications from end devices to remote Web servers.

Yoon et al. [50] proposed a seismic data reconstruction model through recurrent neural network (RNN) algorithms. The authors made tests of different RNN algorithms via the traces to trace approach using available field data provided by a petroleum geo-services company. The ML model training split the training data and validation sets. The proposed model learns high-level features from complex field data and predicts the missing traces in the sparsely seismic sample. A simple comparison of deep bidirectional with and without skip connections was made, using architectures and hyperparameters for both models.

- Seismic data standards.

Suarez et al. [53] described the structure and goals of an integrated system of networks within the International Federation of Digital Seismograph Networks (FDSN), as well as the instrumentation characteristics, data exchange of high-quality seismological data, standardization format, and access. Detrick et al. [54] summarized Global Seismographic Network (GSN) data that are used for the research of operational missions of the USGS, the NOAA, and the Comprehensive Nuclear Test Ban Treaty, as well as studies of earthquakes, tectonics, and volcanology.

Pueyo et al. [57] proposed a communications system, LoRaMoto, which aimed to exchange information about civilians’ safety aftermath of an earthquake when outages in communication networks following earthquakes limit the capacity to obtain information. LoRaMoto helps to extend the LoRaWAN architecture and implements a packet forwarding mechanism to keep emergency management organizations informed. The LoRaWAN network protocol has scalability and performance limitations when there is node mobility. However, the LoRaMoto system does not use node mobility; it is closer to an ad hoc network. A performance evaluation was made by simulating a realistic environment to understand scalability and portability. Limitations in scalability were related to the density and capacity of gateways for node communication.

Ebel et al. [18] presented a description of a seismic monitoring network (RSN—U.S), transmission media, data processing, and collaboration with other organizations to improve the monitoring technology. Its main products have been active structure/fault monitoring, use of earthquake focal mechanisms, and classification of event types. M.Filippucci et al. [59] made a description of the OTRIONS seismic network waveform database, their cloud infrastructure for acquisition, and a storage system with access to the station metadata. Their network had a high level of security in data exchange through multi-protocol VPN services. Yu. E. et al. [55] summarized the main station information system (SIS) features that are a repository for managing, checking, and distributing high-quality metadata. Data centers, such as the Advanced National Seismic System (ANSS), use SIS information to identify parameters such as the overall response, channel gain, and hardware components.

Krischer et al. [43] developed an Adaptable Seismic Data Format (ASDF) to store any number of synthetic, processed, or unaltered waveforms in a single file, including comprehensive meta-information (event or station information) in the same file. Guimaraes et al. [58] analyzed the main file structures for storing and processing seismic data in the cloud and proposed a solution that can improve real-time performance using classic standards (for example, SEG-Y) and modern formats (for example, SEG-Y and ASDF). It decreased seismic processing and helped to efficiently convert to and from SEG-Y.

- Early warning trends.

Behr et al. [60] presented an application of the virtual seismologist (VS) algorithm for earthquake early warning (EEW). A VS algorithm was used to estimate magnitudes and ground motion in the Swiss Seismological Service and other European networks. Perol et al. [61] proposed an algorithm optimization tool for earthquake detection and localization based on convolutional neural networks (ConvNetQuake) for reviewing the exponential growth of the volume of seismic data. This allowed rapid earthquake detection and location for EEW. Tariq et al. [62] proposed a real-time EEW event detection algorithm (SWEDA) that detects seismic wave types, using time and frequency domain parameters mapped on a 2D mapping interpretation scheme. Chin et al. [69] proposed an EEW model through recurrent neural networks (RNNs) for earthquake detection with a real-time response.

The data set included 128 earthquake events collected in the Taiwan zone with 1797 seismic waveforms cut from the earthquake events. Two types of architectures were used, a common model to detect the P-wave and the S-wave characteristics, and a developed model was used to detect three targets: (1) a vector related to a number of input features, (2) LSTM cells to build the hidden layers as storage to preserve the state instance, and (3) the output layer to calculate the final probability for each category of the target events.

Bai et al. [64] applied compressive sensing (CS) to achieve high-efficiency data observations through seismic waveform sparseness, random sampling of observations, and data recovery of seismic waveform data. The model used two conditions: a sparse representation of data in a transform domain, and incoherence between the sampling method and sparse transform. Moreover, other authors such as Baraniuk et al. [65] used CS for digitizing signals and used more general and random test functions processed via measurements. This allowed for faster capture, sampling, and processing rates, with lower power consumption, especially in cases of larger and higher-dimensional seismic data sets. Arrais D. et al. [67] presented a review of the current information availability at seismic monitoring systems. The proposed solutions at software and network infrastructure use data recovery mechanisms through traffic control points in primary nodes and redundancy in data transmission networks to increase information availability. Dimililer et al. [72] presented an overview of IoT models, deep learning and machine learning studies for EEW, and geophysical applications. The study suggested combination techniques for high-resolution seismic imaging based on deep learning algorithms.

Zhang Qi et al. [68] proposed a system of real-time earthquake detection by monitoring millions of queries related to earthquakes from an online search engine (China). The testing set was set up with the results of the MID detector (multi-internal derivative-based detection algorithm) and labelled with earthquake catalogs. Yin et al. [70] developed a KD tree application for large databases to reduce EEW delays identified in the processing time and estimated real-time earthquake parameters. An offline test was made using a database with feature sets of the waveform, and it was compared with real observed seismic parameters. The database was focused on values of peak ground acceleration, velocity, and displacement (PGA, PGV, and PGD), instead of common parameters such as hypocenter distance. Torky et al. [66] used hybrid convolutional neural network (ConvLSTM) techniques to indirectly predict seismic response of mid-rise and high-rise buildings in earthquake-prone areas, and to assist earthquake early warning systems. They used accelerometer mesh with a sampling frequency of 10-20-100 Hz for torsional vibration and waveform detection. For it, some parameters and filtering techniques were applied (including the fast Fourier transform (FFT) Butterworth filter and discrete wavelet transform (DWT) decomposition).

DeLaPuente et al. [71] proposed a seismic simulation workflow to deliver accurate short-time reports of earthquake effects. The objective was to reduce high computational resources for simulations in detailed geological models used in the impact evaluation of large earthquakes. It contains four subsystems deployed as services to produce ground-shaking maps and useful information for civil protection agencies. The simulation procedure contains an automatic alert service, smart access, a control center, and a post-process service. Korolev et al. [63] proposed an automated information system (AIS) for data processing in a specific geographic area for observation science data integration. Their main characteristic was the homogeneity of the instrumental network through Reftek Sw/Hw, the RTPD protocol, and the Zabbix monitoring system.

Within the methodology used by several authors, it was possible to identify the mechanisms or platforms, protocols, formats, and topologies. Furthermore, these evaluation parameters applied to the selected articles were contrasted with four application areas, and, as a result of this analysis, the next section contains the most relevant contributions from reviewed studies.

## 3. Results

**Figure 5:**
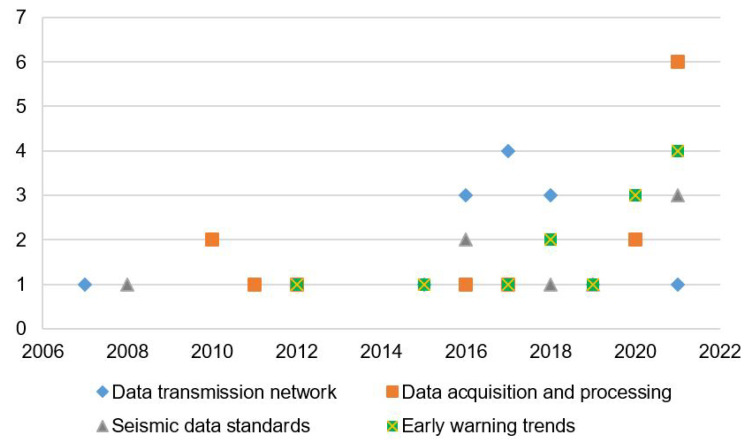
Year of publication per applications areas.

This research focuses on identifying factors that may affect the availability of the information required by data centers for early warning diffusion on seismic and volcanic phenomena. Therefore, this section reviews relevant studies related to four fields: (a) data transmission networks, (b) data acquisition and processing, (c) seismic data standards, and (d) early warning trends. However, it is essential to mention other, yet no less important, processes that involve the communication system of these organizations. These are data storage, analysis, and diffusion of information for subsequent research in geophysics, geology, seismology, and volcanology. Each of these fields was compared through an individual analysis, obtaining summaries described in Table 1, Table 2, Table 3 and Table 4, with methods, characteristics, application areas, and approaches. Figure 5 shows the distribution by year of the publications that are reviewed in this study and distributed by application areas.

From selected articles, most of these date from 2015 to the present. The trend shows that between 2016 and 2021 seismological organizations have emphasized improving seismic data transmission by including quality parameters and an evaluation of seismic networks such as [26,31,36]. In the same way, for the areas of data acquisition and processing, as well as early warning trends, there are many studies which demonstrate a rising trend over the last 4 years, such as [43,46,49]. The application and acquisition of information systems have been associated with the determination of early warnings to help with the detection of earthquakes and seismic location, the evaluation of magnitudes, and use of seismic data with other SDC, among others. On the other hand, the formatting trend for seismic data processing is more constant, because seismic data standards have reached maturity and global diffusion. Therefore, they are used by most seismological networks worldwide. The main objective is the exchange of data between these global networks. Several of the selected studies [18,55,58] show that seismological communities have implemented protocols and software solutions to unify data acquisition systems and their compatibility in the last decade. However, data processing methods have been unified to a lesser extent; these solutions are ad hoc in most cases.

### 3.1. Data Transmission Networks

Instrumental networks for seismic and volcanic monitoring have different sensor types (e.g., seismic sensors, accelerometers, inclinometers, high-precision GPS, monitoring cameras, pressure sensors, etc.) and components (e.g., data acquisition systems, data loggers, transmission media, energy sources, physical infrastructure, etc.). They bring seismic information to seismological data centers to monitor seismic and volcanic events in real time. The following articles are related to the research questions proposed in this study and deal with seismic data transmission and seismic networks. The information is compiled in Table 4 and presents a summary of the most important findings.

Regarding latency reduction in communication networks for earthquake early warning (EEW), the authors [23,24] identified that some data could be improved by deploying software upgrades and setting the data-logger parameters. In addition, they succeeded in recording latency data per packet in a MSEED (SEED, Standard for the Exchange of Earthquake Data) (MSEED contains raw waveform data, but does not include metadata) format, as traditionally performed with seismic waveform data. This has allowed them to identify the performance of telemetry links in real time. In future work, they suggest developing data-logger algorithms, which reduce data latency by transmitting final solutions or light seismic parameters instead of continuous data that respond to data transmission limitations toward data centers in case of strong earthquakes. Regarding the compatibility of formats for data transmission, [26] has mentioned the advantages of using Dataless SEED to share metadata. Furthermore, the recent Extensible Markup Language (XML) FDSN format helps with metadata usage and compatibility with other formats.

Regarding data compression in data transmission, Li, Huailiang et al. [25] used the Lempel–Ziv–Markov chain algorithm (LZMA) and the deflate algorithm to decrease the size of SEG-Y files, and, in the experimental results, showed that the improved algorithm could provide better compression and compatibility than algorithms of standards established by the Geophysical Exploration Society (SEG) and the Steim2 compression algorithm proposed by the Standard for the Exchange of Earthquake Data (SEED). Helal et al. [36] used a convolutional neural network (CNN) focused on a low-compression ratio (CRs) and a signal-to-noise ratio (SNR) to improve high and moderate seismic data CRs. Their CNN model used Keras with TensorFlow backend and Google COLAB GPU. Results improved bandwidth in communication links and storage media.

Regarding the improvement in systems or complete networks, Scarpato et al. [27] have proposed a tool to improve the performance of the data transmission system of the real-time monitoring network for the surveillance of a specific volcanic area (Campi Flegrei, Italy). The authors’ contribution helped increase transmission speed, network availability, and connection reliability, which together guarantee continuous monitoring and fault tolerance. For network quantification, the authors used performance, delay, standard deviation, and packet loss statistics. The quality parameters established were: real-time delay (msec. transmission), availability (continuous monitoring in real time), robustness (tolerance to faults) in transmission, acquisition, and visualization. The mentioned system has allowed review of network conditions, as well as activate, execution, and repair operations such as ping (lost packets and delays), throughput test (average throughput, network performance, change of frequency, and channel), and node check (periodic operations to detect and restore point-to-point links). These actions are recorded in reports and feedback in a MySQL database. On the other hand, the system has allowed review of seismic network conditions with the Earthworm seismic data acquisition system. The author quantified the effectiveness of the monitoring system by relating the number of failures in an observation interval with the average response time to repair them; with favorable results, they were able to improve availability by a 4.8%.

Studies related to the instrumental network structures for seismic–volcanic monitoring and solutions implemented in data centers [28,29] have proposed improvements for the autonomy of seismic equipment, mainly in terms of energy consumption and local storage. Other authors have proposed solutions for data acquisition systems, as well as arrays of sensors, digitizers, and wireless transmission designed to reduce energy consumption, autonomy, and extended operation [30].

Regarding efficient routing protocols, Y. Zhou et al. [31] designed a routing protocol to reduce power consumption and latency using opportunistic routing. The sender selects the suitable ones from a group of neighboring nodes with specific criteria (priority of Q-maximum value, routing timeout for UWSN, and multipath suppression) for forwarding packets to the next hop with optimal routing. The results were obtained by mathematical analysis and simulation. Although the performance of this protocol was compared with other Q-learning protocols, any-path routing protocols based on “greedy approaches” do not consider long-term end-to-end rewards.

Other studies have highlighted MAC protocols based on TDMA and subsequent comparisons [32,33]. These are mainly improvements for wireless sensor networks in power consumption, latency, bandwidth, and network configuration topology (energy consumption, scalability, delay, traffic adaptability, throughput, and overheads). Iqbal et al. [34] used WSN analysis and Markov Chain models related to the network performance, transmission time from the nodes to the gateway, power consumption, scalability, QoS, redundancy, and failure tolerance.

In reference to data collection and efficient data transmission, Mothku et al. [35] proposed a mechanism for reliable data transmission applied to WSN, considering link loss rates and redundancy. A Markov decision process (MDP) identifies the level of packet redundancy to improve reliable data collection and decrease the rate of data transmission. Moreover, it analyzes the link state quality and considers other parameters such as the number of data transmissions, the average delivery delay, energy consumption, and network lifetime. The results obtained were used through experimentation. These advances could be applied in the future as an effective solution in environmental monitoring, tsunami alerts issued, or seismic sensor networks for real-time monitoring.

Regarding common network access control standards used, Zhong et al. [38] focused on solutions for low-power consumption and stable communications through the use of standards such as IEEE 802.15.4 between nodes to ensure low-speed and IEEE 802.11b/g network structures for connection between clusters to ensure high data transmission rates. Reddy et al. [37] used IEEE 802.11ad standards to simulate a seismic network with an efficient data transfer scheme for seismic acquisition with gigabit rate requirements to subsurface imaging with high quality and superior depth. The main objective was to reduce the energy consumption in a seismic network with a high number of hops, sacrificing latency.

### 3.2. Data Acquisition and Processing

Regarding data processing, in Table 5 below presents the most important findings of data acquisition and processing related to the proposed research questions. From this compilation, we could highlight several studies, as indicated below:

In a real-time monitoring network, the seismic data acquisition comprises data arrival to the seismological data centers for subsequent storage, processing, and interpretation. Generally, data centers use acquisition and processing systems compatible with different seismic data formats by monitoring stations, such as SEED, MSEED, SAC, CD1.1, ASCII, SEG-Y, PH5, SEISAN, SUDS, GEOCSV, and others [16,39], as argued by Ringler et al. [26]. These formats contain data representing the recording of sensors on different channels (seismic time series data) and control headers (time UTC, identifier code, channel, others). Acquisition systems allow finding essential parameters for event locations (joint hypocenter determination, moment tensor, focal mechanism, magnitude, seismic moment, depth, intensity, inversion, and others). These parameters are subsequently validated semi-automatically by relating recognized important events with other parameters associated with the phenomenon, such as teleseism, regional earthquakes, satellite images, volcanic observatories monitoring, cameras monitoring, infrasound detection networks, meteorological phenomena, community reports, among others [1,73].

Regarding filtering methods to reduce seismic noise caused by site effects, ambient noise, or data gaps. Solutions based on the relevance vector machine (RVM) method are applied to seismic signals to decrease filtering effects and gaps in seismic waveforms, as shown by aliasing [41]. This allowed greater effectiveness instead of applying common algorithms with the compressive sensing (CS) framework for data reconstruction that did not consider uncertainty quantification or feature learning. Other methods used were Hankel sparse low-rank approximation [47], where the attenuation of seismic signal noise in data processing was probed as the main factor that affects the accuracy and quality of final seismic images and weakens complex traces.

Other studies have developed ad hoc software tools to improve seismic data analysis and data processing. With the adoption of different formats (SEED, SAC, ASCII, Y-Nanometric, GSE, and others), these solutions should be adaptable to other seismology applications [40,42]. Furthermore, these solutions helped to cover functionalities for waveform analysis, such as genetic algorithm, least-square fitting, auto-picking, fast Fourier transforms, location, attenuation, focal mechanisms, waveform, modeling techniques for rapid estimation of earthquake source parameters, and others. Other authors [51] used deep convolutional neural networks to convert geological project files to a data format suitable for deep learning with processing, analysis, model evaluation, and comparative results. The main disadvantage is a large amount of data for training and automatic seismic interpretation.

Furthermore, [44,56] have proposed a framework for processing seismological data in the ObsPy Python library for seismology geared toward processing, and specifically on-time series analysis with file compatibility and conversion formats. Moreover, ObsPy can save fragments of data separately, which reduces the overlaps produced by clock time lag during data recording, as well as the gaps caused by interruptions in data transfer [45]. The functionality of the ObsPy library has also been mentioned in the processing of data and metadata for seismic signals, as well as the graphical user interface [46]. However, the ObsPy functions are used to complement post-acquisition and analysis, and it is not focused on data analysis in real time.

Regarding methods to reduce seismic data processing time, unsupervised machine learning processes were designed [48]. For this purpose, seismic data were converted into 3D dispersion images. Methods used were Gaussian mixture model (GMM) clustering for dispersion image points and background noise points. Then, the density-based spatial clustering of applications with noise (DBSCAN) algorithm allowed discrimination modes of dispersion energy points. Noise interference was reduced with a particle filter to smooth the picked dispersion curves. Other solutions were focused on open-source software development using deep learning models [49]. The goal was to improve the accuracy of the seismic waveform arrival detection system and to reduce the manual selection procedure for seismic phase arrival times. The method applied for automatic seismic wave and phase detection was based on the PhaseNet neural network.

Regarding seismic data reconstruction, several processes point to convolutional neural networks, based on computer vision and imaging processing to identify bad or missing traces, which can cause problems for seismic data processing. However, ML techniques and (RNN) algorithms perform seismic trace interpolation. As an alternative solution [50], the neural network (RNN) algorithm model was used to identify sequences of time-series data through deep bidirectional long short-term memory (LSTM), and test models with and without skip connections. However, the proposed model was only tested on a simple experimental combination of input and output traces for prediction and interpolation.

### 3.3. Seismic Data Standards

Recently, international seismological communities through global seismic networks exchange information with emphasis on the standardization and quality of seismic data by applying data sharing policies for scientific purposes and early warnings worldwide monitoring [53,54]. For example, FDSN promotes free and open access to seismological data for the benefit of scientific research and disaster prevention and mitigation. Worldwide networks such as GSN have the main advantage of the globally distributed network size, which provides robustness, uniform high-quality, broadband capabilities, and high-dynamic range recording of ground motion, among others. Formats most used by seismological data centers are SEED, MSEED, SAC, CD1.1, ASCII, SEG Y, PH5, SEISAN, SUDS, Reftek, GEOCSV, QuakeML, XML, and others, mainly with the use of standard Seedlink and Arclink protocols. With the aforementioned, Table 6 shows the compilation of the most relevant aspects of seismic data standards concerning the proposed research questions.

Several studies have presented important features of current seismic monitoring networks, transmission media, data processing, and other products (e.g., regional seismic networks (RSNs) of ANSS-USGS) [18]. Other descriptions have highlighted the capabilities of seismic networks with cloud infrastructure for acquisition and as a storage system with access to the station metadata [59]. Another relevant aspect has been the use of VPN services (Mikrotik’s RouterOS) based on SSTP for seismic stations, the VPN tunnel (OpenVPN) for the user, and the VPN tunnel (PPTP) for the Seiscomp3 system to improve access security and information exchange. In another study [55], the importance of high-quality metadata for successful operations of seismic networks and research through links to other external repositories has been considered. Metadata provides information on data inventory, instrument responses, installation data, calibration, maintenance, and instrumental responses. Moreover, common standards used include FDSN StationXML and dataless standards for the exchange of earthquake data. It is important to note that the information contained in metadata is not included in the Standard for Seismic Data Exchange (SEED) or in the StationXML format of the FDSN. These standards only contain partial information on the seismic station, instrumental response, and network.

It is relevant to recognize that several organizations (e.g., USGS, IRIS, IASPEI, CTBTO, FDSN, GEOSS, and others) have participated in the seismic data standardization process over the last 30 years [39]. Currently, applications for the use and conversion of these formats continue to improve the exchange of information between seismological communities [43]. Additionally, current studies pretend to include comprehensive metainformation (event or station information) in the seismic files that could support standards such as Quake Markup Language (QuakeML), StationXML, W3C PROV, and HDF5. Moreover, it is suggested that applications should be developed on Python, and tools coupled to ObsPy.

### 3.4. Early Warning Trends

The articles mentioned below deal with early warning trends and are related to the research questions proposed in this study. Table 7 presents a compilation of the most relevant aspects. Commonly, seismological data centers use open-source earthquake monitoring systems (e.g., Seiscomp3) for seismic data acquisition; this improves the compatibility with traditional analysis methods and these data centers require very accurate real-time results even when the network is not advanced.

Some studies have proposed improvements in determining earthquake early warnings [60,61,62,69], optimized algorithms, and recurrent neural networks for magnitude estimation and earthquake detection. One particular solution focused on Seiscomp3 compatibility based on the Bayesian approach and used envelopes of acceleration, velocity, and displacement as the basic data input. Another study contributed to computational performance improvement and memory usage. However, we noted the requirement of a large training set for good earthquake detection and location performance. Other studies considered gradient mapping, auto-calibration, seismic noise filtration (SNF), peak detection sequence, scaling coefficient arrays, STA/LTA arrays, and earthquake probabilistic sequence for algorithm development. In addition, other results showed a disadvantage of traditional methods for earthquake detection waves and empiric criterion, which result in false alarms decreasing the credibility of the system, allowing simulation results as well as the identification of better performance compared to traditional schemes in terms of detection, accuracy, and processing time.

Studies on compressive sensing (CS) processing techniques for efficiently acquiring and reconstructing a signal were applied in [64,74]. When it was sampled significantly below the Nyquist rate, it could retrieve data by random sampling on the signal structure in measurements that appear to be "noise" interference. Later, it became possible to reconstruct a coherent signal. The CS method demonstrated efficiency in image resolution, compression, adaptability, and time reduction in seismic data acquisition. Another study [65] proposes CS using random functions to combine Shannon–Nyquist frequency sampling with large-scale optimization on sparse structure signals for signal recovery and processing. The aim is to use larger data sets, speed in processing, lower bandwidth and acquisition time, and energy consumption reduction. Another advantage of CS capability was sampling below the Nyquist rate, sensing by dimensional reduction, preserving measurements, signal recovery, and reconstruction from real data. In addition, CS uses compression algorithms to transform the signals to another domain (DCT and wavelet transform). However, CS could face challenges in seismic acquisition related to the representation of variable seismic noise in different monitoring sites, sampling precision, efficiency in signal reconstruction algorithms, and signal–noise ratio.

Other authors presented a review of the methods for data processing, storage, and availability of information used in volcanic seismic monitoring centers, as well as trends to decrease data gaps identified due to limitations of the acquisition systems and transmission networks, leading to possible errors in the quantification and interpretation of seismic monitoring in real time [67]. Furthermore, a review of IoT models, deep learning, and machine learning algorithms for EEW and geophysical applications summarized methods for seismic imaging [72]. Furthermore, convolutional neural networks could be applied to seismic wave simulation, velocity prediction, and density profiles used in earthquake detection, EEW, seismic tomography, and earthquake geodesy.

Regarding EEW delays, researchers have developed techniques to reduce the delay time between earthquake detection and the final information delivered to the civil population. Some approaches monitor an online search engine in order to respond quickly to a large number of users making relevant queries, exploiting search queries as effective “crowd sensors” for event monitoring [68]. Other approaches have constructed a KD tree organized by nearest neighbors (NNs) which reduces the search time in a large data set during ongoing earthquake records [70]. Deep learning techniques (ConvLSTM) have also been used to indirectly predict the behavior of seismic events with time–frequency analysis of the acceleration response and filtering of time series data with discrete wavelet transform [66]. All of these efforts can reduce the warning delivery time during a seismic event.

On the other hand, regarding efficient computational resources, studies have proposed minimizing the time taken to make accurate earthquake reports [71]. Some approaches reduce the simulation workflow in seismic events using (HPC) super-computational resources to enable the execution of automatic warning service, intelligent access, control centers, and post-processing services by automatically collecting information on possible earthquakes and also validating 3D simulations or processing new information for simulations. Other authors have developed algorithms and software tools to homogenize instrumental seismic networks by adapting modules for acquisition, preprocessing, and data storage, allowing information systems to better integrate information for research and monitoring of natural hazards in a specific geographic area [63]. With the efficient use of computational resources, the workflow to deliver accurate reports of seismic events is accelerated and the delivery time to the end user from the occurrence of the earthquake is shortened.

## 4. Discussion

The results presented in Section 3, after applying the methodology described in Section 2, have allowed us to identify the current mechanisms, techniques, methods, standards, and trends that support the availability of information in acquisition and processing systems. We can more precisely answer the research questions proposed in Section 2.1 based on different topics recognized in our results. In addition, this section elaborates on related works and contributions in this paper.

### Research Question Analysis

- Other studies have focused on developing specific systems to guarantee the availability of information in a seismological network’s rebuilding waveform with buffered data. Nevertheless, they do not represent solutions applicable to other networks, in response to RQ1. Moreover, these solutions require a network infrastructure that allows physical redundancy and redirection of data traffic through an alternative route, but at high costs and greater complexity.
- Global Seismographic Networks allow information management from affiliated seismological networks. They also provide reliability, as well as quality of service in the acquisition, processing, data storage, and analysis of information for early warnings. However, they focus on providing transmission media that guarantee the stability of data arrival for specific stations only, which will be part of the global monitoring network. Therefore, it is a partial solution for RQ1 and RQ2 because it requires exhaustive processes for the certification of seismic stations that will be members of global networks, which do not include the total of local networks for specific regional monitoring. This is useful for strong earthquake detection. On the other hand, in this case, the diversity of monitoring stations is not considered either.
- It is important to note that the information contained in the metadata is not included in the seismic data formats and standards for seismic data exchange. However, the information contained in those archives could contribute to the identification of failures in instrumental networks. Therefore, as one of the solutions to RQ2, it is possible to include metadata in real-time data acquisition and processing systems. This will decrease interruptions or failures in the monitoring stations and may contribute to improving the availability of information.
- Some studies have identified fundamental advances related to data compression algorithms. Some of them could help to send information employing transmission media with limited capacity. However, these possible solutions for RQ2 may apply to specific formats only, and may require additional mechanisms for acquisition systems and additional format conversion processes in relation to their computational cost.
- Solutions to improve the availability of information based only on investment in the infrastructure for satellite transmission and external services may require very high monetary costs because the instrumental networks comprise hundreds of monitoring points. Furthermore, this does not guarantee a nearby backup channel, and the seismological data center will probably require an internet link managed by outsourcing services. As a consequence, it shows a vulnerability in the availability of the information due to the dependence on external service providers and backup connections. With the aforementioned, RQ3 can be partially answered because the use of satellite communications for real-time seismic monitoring is one of the most recent alternatives. However, several seismological centers do not have sufficient financial resources to implement these services.
- Latency in monitoring networks can be reduced by software upgrades in data-loggers and setting seismic equipment. However, this partial solution for RQ3 does not apply to every manufacturer’s brand of data-loggers. Moreover, compatibility with software versions may need to be checked.
- Through Obspy Python, multiple functions have been developed for the processing and reconstruction of seismic data. These solutions are applied in post-acquisition and processing. This may improve the compatibility of formats. Therefore, these frameworks could be used to develop seismic data recovery mechanisms. With Python functions, it is possible to contribute to reduced seismic data gaps in the acquisition and processing systems of seismological data centers. Therefore, in response to RQ2 and RQ3, several of the related works detailed in Table 5 can be used to consolidate a new solution that includes access to monitoring networks and transmission media.

## 5. Conclusions

This study has identified some factors that affect the availability of information in seismic–volcanic monitoring systems. Therefore, proposed solutions at the software level and network infrastructure may focus on data recovery mechanisms through traffic control points in primary nodes. Additionally, a new protocol may be developed to add redundancy in data transmission networks and increase the availability of information in acquisition and processing systems.

In this literature review, we mentioned that several studies applied new methodologies such as machine learning [31,41], deep learning [49,66,72], convolutional neural networks [36,61], among others. Mainly, they have focused on the processing and post-processing of feature data and the interpretation of seismic signals. However, we are mainly looking for files or packets that correspond to information gap characteristics, which can be identified by values close to the instrument’s reference noise response value in each channel during the seismic data frame arrival sequence. In addition to this, values above the conversion thresholds of data-loggers and acquisition systems. Another specific objective of this study will be creating a database that gathers these gap identifiers, finds their correlation, and thereby applies an algorithm for information retrieval on demand. In other words, it can be pre-established by user requirements in SDCs.

This literature review has demonstrated that this issue has not yet been considered as a comprehensive proposal or solution applicable to instrumental networks interacting in transmission media, acquisition, and processing systems for seismic and volcanic monitoring centers. There are also no specific solutions to recover data gaps caused by instability or interruption in seismic–volcanic monitoring networks. Without the information available, all the scientific advances for acquisition and processing that have been developed are not effective in reducing response times to early warning emissions during data acquisition and processing. Therefore, we presented research that can supply strategy development to improve the availability of information in seismic–volcanic networks.

As a result of this work, it has been identified that the loss of information that occurs due to vulnerabilities between instrumental networks and acquisition systems has not been considered an important issue. A comprehensive method has not yet been found to solve seismic and volcanic monitoring problems related to data failures, breaches, transmission interruptions, and others focused on information security strategies to increase the availability of data acquisition and processing systems in different SDCs. This previously mentioned issue is due to an ideal communication system that guarantees information availability. Currently available studies allude to the significant investment and resources in the network infrastructure to deal with delays during the transfer and recovery of information.

Therefore, information security mechanisms based on algorithms and protocols which focus on increasing availability in instrumental networks offer a novel approach to enhance acquisition and processing systems. Moreover, suggested mechanisms may contribute to the optimization of current network management, diagnosis, and failure identification systems. As a consequence, the time response in the early warnings of seismic and volcanic events can be reduced. Finally, the implementation of this approach can reduce operational costs related to semi-automatic processes for data recovery.

In future works in light of this review, the construction of a data set has been proposed that contains a classification of files obtained from monitoring stations of a real network made up of different sensors of speed, acceleration, deformation, geochemistry, and visualization cameras with continuous data in 3 months of the year 2021. The goal is to find information gaps in frames using sequence file characterization algorithms and to develop data recovery mechanisms by evaluating enabled transmission media and redundant links to monitoring points (stations). Subsequently, the proposed model should be tested in an isolated environment from a real seismic–volcanic monitoring network through secondary nodes and a central synchronization node with the acquisition systems of the primary data center. Finally, data recovery and the improvement in the availability of information from the data centers should be evaluated, as well as the contribution to early warnings in seismic–volcanic monitoring.

On the other hand, as a perspective to the proposed information retrieval method results, techniques based on artificial intelligence could be applied to automate these processes in the future. With the analysis of this study’s results, it may be possible to develop automatic recovery functions through machine learning algorithms and validation methods that reduce the response time of early warnings to seismic and volcanic events.

## Figures and Tables

**Table 1:** Structuring search strings.

Most Important Terms	Search Expression
RQ1	
*algorithms and protocols related to availability of information*	seismic data (AND) algorithms (OR) protocols (AND) data acquisition
	seismic networks (AND) algorithms (OR) protocols (AND) availability
	real-time seismic data (AND) algorithms (OR) protocols (AND) seismic networks
RQ2	
*standards, formats, and systems for seismic data acquisition and processing*	seismic data (AND) formats (OR) standards (AND) acquisition (OR) processing
	seismic monitoring networks (OR) volcanic monitoring networks (AND) formats (OR) standards (AND) acquisition systems (OR) processing systems
RQ3	
*seismic–volcanic monitoring trends for seismic data transmission and redundant seismic networks*	seismic monitoring networks (OR) volcanic monitoring networks (AND) data transmission (AND) redundancy
	seismic monitoring networks (OR) volcanic monitoring networks (AND) redundant systems (AND) early warnings

**Table 2:** Summary of search results.

Research Question	Search Expression	Scopus	ScienceDirect	ACM	IEEE Xplore	Total
*RQ1*	seismic data AND data acquisition AND (algorithms OR protocols)	40	168	124	91	423
	seismic networks AND availability AND (algorithms OR protocols)	47	86	31	5	169
	real-time AND seismic data AND seismic networks AND (algorithms OR protocols)	252	85	13	61	411
*RQ2*	seismic data AND (formats OR standards) AND (acquisition OR processing)	55	63	140	161	419
	(seismic monitoring networks OR volcanic monitoring networks) AND (formats OR standards) AND (acquisition systems OR processing systems)	14	142	13	13	182
*RQ3*	data transmission AND redundancy AND (seismic monitoring networks OR volcanic monitoring networks)	47	94	25	-	166
	redundant systems AND early warnings AND (seismic monitoring networks OR volcanic monitoring networks)	15	109	43	1	168
Total:		470	747	389	332	1938

**Table 3:** A summary checklist for quality assessment.

	Article (Author)	Q1	Q2	Q3	Q4	Q5	Q6	Q7	Q8	Q9	Q10
Data Transmission Networks	A1, Behr et al. [23]	✓	✓	✓	✓	✓		✓	✓	✓	
	A2, Stubailo et al. [24]		✓		✓	✓		✓	✓		✓
	A3, Li et al. [25]	✓	✓			✓		✓	✓		✓
	A4, Ringler et al. [26]	✓	✓	✓	✓	✓	✓	✓	✓	✓	
	A5, Scarpato et al. [27]		✓			✓		✓	✓		
	A6, Vidal et al. [28]	✓				✓	✓	✓	✓	✓	
	A7, Weber et al. [29]	✓				✓	✓	✓	✓		
	A8, Adams et al. [30]	✓				✓	✓	✓	✓	✓	
	A9, Zhou et al. [31]	✓	✓		✓	✓	✓	✓	✓		
	A10, Kaur et al. [32]	✓			✓	✓			✓		✓
	A11, Piyare et al. [33]	✓			✓	✓			✓		
	A12, Iqbal et al. [34]	✓			✓	✓		✓	✓		
	A13, Mothku et al. [35]	✓	✓		✓	✓	✓	✓	✓		
	A14, Helal et al. [36]		✓	✓	✓	✓	✓		✓	✓	
	A15, Reddy et al. [37]	✓	✓			✓			✓		
	A16, Zhong et al. [38]	✓	✓			✓	✓	✓	✓		
Data Acquisition and Processing	A17, Dost et al. [39]	✓	✓	✓	✓	✓		✓	✓		
	A18, Cordery et al. [40]		✓		✓	✓		✓			
	A19, Pilikos et al. [41]	✓	✓	✓	✓	✓		✓	✓	✓	
	A20, Abdelwahed et al. [42]	✓	✓	✓	✓	✓	✓	✓	✓	✓	
	A21, Krischer et al. [43]	✓	✓	✓	✓	✓	✓	✓	✓	✓	
	A22, Beyreuther et al. [44]	✓		✓		✓	✓	✓	✓		
	A23, Megies et al. [45]	✓	✓	✓		✓	✓	✓			
	A24, Hosseini et al. [46]	✓	✓	✓	✓	✓	✓	✓	✓	✓	
	A25, Anvari et al. [47]	✓	✓	✓	✓	✓	✓	✓	✓		
	A26, Wang et al. [48]	✓	✓	✓	✓	✓	✓	✓	✓		
	A27, Zhao et al. [49]	✓	✓	✓	✓	✓	✓	✓	✓		
	A28, Yoon et al. [50]	✓	✓			✓	✓	✓			
	A29, An Y. et al. [51]	✓	✓		✓	✓	✓	✓	✓		
	A30, Bin et al. [52]		✓			✓	✓	✓	✓		✓
Seismic Data Standard	A31, Suarez et al. [53]	✓	✓	✓	✓	✓	✓	✓	✓		
	A32, Detrick et al. [54]				✓	✓		✓	✓		✓
	A33, Ebel et al. [18]	✓	✓	✓	✓	✓	✓	✓	✓	✓	
	A34, E.Yu et al. [55]	✓	✓	✓	✓	✓	✓	✓	✓	✓	
	A35, Krischer et al. [56]	✓	✓	✓	✓	✓	✓	✓	✓	✓	
	A36, Pueyo et al. [57]	✓	✓		✓	✓		✓	✓		
	A37, Guimaraes et al. [58]	✓	✓	✓	✓	✓	✓	✓	✓	✓	
	A38, Filippucci et al. [59]	✓	✓		✓	✓	✓	✓	✓		
Early Warning Trends	A39, Behr2016 et al. [60]	✓	✓	✓	✓	✓	✓	✓	✓		
	A40, Perol et al. [61]	✓	✓		✓	✓	✓	✓	✓		
	A41, Tariq et al. [62]		✓			✓		✓	✓		✓
	A42, Korolev et al. [63]	✓	✓			✓	✓	✓	✓		
	A43, Bai et al. [64]	✓				✓		✓	✓		
	A44, Baraniuk et al. [65]	✓	✓			✓	✓	✓	✓		
	A45, Torky et al. [66]	✓	✓	✓		✓	✓	✓	✓		
	A46, Arrais et al. [67]	✓	✓	✓	✓	✓	✓	✓	✓		
	A47, ZhangQi et al. [68]	✓	✓	✓	✓	✓		✓	✓		
	A48, Chin et al. [69]	✓	✓	✓	✓	✓	✓	✓	✓		
	A49, Yin et al. [70]	✓	✓	✓	✓	✓	✓	✓	✓		
	A50, DeLaPuente et al. [71]	✓				✓	✓	✓	✓		
	A51, Dimililer et al. [72]	✓				✓	✓	✓			

**Table 4:** Data Transmission Networks—Results.

Section	Data Transmission Networks	
**Article**	A1,A2,A3,A4,A5,A6,A7,A8,A9,A10,A11,A12,A13,A14,A15,A16	
**Focus **	EEW algorithm, testing and evaluation. Data delivery latency improvements. Compression algorithm for seismic data transmission. Structure of SEED format. Seismic network description and infrastructure. Energy consumption and local storage improvement. Routing protocol UWSN, WSN, seismic application	
**Methods used in experimental analysis **	**Mechanism**	Virtual seismologist VS(SC3). OnSite algorithm on data-loggers, avoid collisions, compression algoritms comparison, TDMA protocols comparison, miniSEED, and dataless standard. Quality parameters: real time, availability, robustness. Software SEISAN, MATLAB, RINEX data format using TEQC software. (QLFR) protocol to prolong lifetime.
	**Protocols**	TCP/IP, DART and NET, NSFNET. TDMA-based MAC protocols, depth-based routing (DBR), QLFR using Q-learning algorithms, protocols focused on beam routing (FBR), common encoding types STEIM 1 and STEIM 2 compression. Routing protocols WSN, IEEE.(802.11-802.11.4-802.15.4), ArcLink protocol (SeisComp3), RefTek RTPD server, vector-based forwarding (VBF), SeedLink protocol in data transmission.
	**Formats**	QUakeML, SEEDLink, SEG-Y, mSEED. Algorithm: Deflate, Steim2 compression, BZip2. Common channel-naming conventions and focal mechanisms: FMHASH software. GUI of Jiggle software (ISTI/AQMS), routing protocol design can be exchanged with data packet transmission. Frame format of TDMA protocol, TDMA scheduling algorithms, Matlab, C++, Java, OPDMAC, Micaz.
	**Topologies**	Seismic networks for EEW, SCSN seismic networks, SEG-Y, MiniSEED data format and distribution, analog and digital seismic networks (UHF, digital radio links, VPNs, WiFi/Hyperlan radio bridges). Regional GNSS, Osiris(Agecodagis) data-loggers, linux Earthworm system. Low-power satellite-timed seismic data acquisition, mobile underwater sensor networks, WSN protocols, tools and simulators, overview of TDMA-based MAC protocol designed for large-scale cluster-based wireless sensor networks.
**Application Area (Scope)**	Systems for seismic early and post-event warning in a regional area. Results from seismic networks using Seiscomp3. Tuning the data-logger parameters, deploying software upgrades. Storage space optimization for data acquisition, decreasing transmission costs, and improving transmission efficiency. Dataless SEED uses: station metadata, instrument response information, and physical location. Seismic network and collaboration between regional networks. Time stamps, non-volatile memories, low-power consumption electronics, and dynamic voltage control techniques. TDMA scheduling algorithms: scheduled entity, network topology information, and entities to produce and maintain the schedules.	

**Table 5:** Data Acquisition and Processing—Results.

Section	Data Acquisition and Processing	
**Article**	A17,A18,A19,A20,A21,A22,A23,A24,A25,A26,A27,A28,A29,A30	
**Focus**	Deconvolution, data processing workflow. Data reconstruction algorithms and acquisition processes. Seismic data formats. ObsPy framework and gaps.	
**Methods used in experimental analysis**	**Mechanism**	Early deterministic deconvolution workflow. SGRAPH system includes generalized ray theory (GRT); genetic algorithm (GA); least-square fitting; auto-picking; fast Fourier transforms (FFT); ObsPy; Python library for seismology; RSAM, RSEM, SSAM and SSEM algorithms; ObspyDMT functionalities; query of station metadata; earthquake source metadata; plot to visualize metadata.
	**Protocols**	SGRAPH supports common data formats, such as SAC, SEED, GSE, ASCII, and Nanometrics Y-format. Relevance vector machine (RVM) and a probabilistic data-driven model. Processing of retrieval data sets, seismological data retrieval tools: support for data exchange protocols FDSN, web services, Arclink.
	**Formats**	Loaded traces are maintained; processed; plotted; and saved as SAC, ASCII, or PS (post script) file formats. Python libraries NumPy and SciPy, SEED data format, XML format, rdseed files, evalresp files, RESP files, WIN files to SAC standard format, FDSN service interfaces (ObsPy): fdsnws-station for accessing station metadata in StationXML format. Wilber, WebDC, NetDC, Breq-Fast, Emerald, IGeoS, SOD, obspyDMT.
	**Topologies**	Maintaining and analyzing seismic waveform data in a stand-alone environment. Format Structure: SEED, SAC, GSE, CSS, SEISAN, miniSEED, ASCII, ESSTF. fdsnws-dataselect for accessing time series in miniSEED format, and fdsnws-event for accessing earthquake parameters in QuakeML format.
**Application Area (Scope)**	Reconstruct seismic data and simultaneously quantify uncertainty. Characteristics of waveform data formats. Python toolbox that simplifies the usage of Python programming for seismologists. ObspyDMT used for query, retrieval, processing, and management of seismological data sets, including very large, heterogeneous, and dynamically growing ones.	

**Table 6:** Seismic Data Standards—Results.

Section	Seismic Data Standards	
**Article**	A31,A32,A33,A34,A35,A36,A37,A38	
**Focus**	FDSN, SEED standard. Global Seismographic Network (GSN). Metadata. Adaptable Seismic Data Format (ASDF).	
**Methods used in experimental analysis**	**Mechanism**	Control headers (ASCII): volume identifier headers, abbreviation dictionary headers, station headers, and time span headers. Large data set for managing metadata. ASDF uses C/Fortran and Python-based APIs coupling to SPECFEM3D GLOBE and ObsPy toolkits.
	**Protocols**	Conversion protocol, compression protocol. Comparison of: SAC, miniSEED, SEG Y, PH5. ASDF libraries for reading, writing, conversion, and visualization.
	**Formats**	SEED, telemetry volume format. Time series data and related metadata. SEED, FDSN, StationXML, dataless SEED, ExtStationXML. Standards such as QuakeML, StationXML, W3C PROV, and HDF5 must resolve efficiency, data organization, data exchange, reproducibility, mining, and visualization and understanding of data.
	**Topologies**	Seismic monitoring networks. These include configuration, firmware, sensor status, calibration parameters, installation information. The HDF5 format allows efficient and parallel I/O operations, integrates compression algorithms, and checks sums to guard against data corruption.
**Application Area (Scope)**	SEED provides a special Telemetry Blockette, transmission of only the newest data. Permanent network globally distributed. The Station Information System (SIS) provides a valuable bridge between field equipment and the end user’s metadata. Inclusion of comprehensive meta information.	

**Table 7:** Early Warning Trends—Results.

Section	Early Warning Trends	
**Article**	A39,A40,A41,A42,A43,A44,A45,A46,A47,A48,A49,A50,A51	
**Focus**	Virtual seismologist (VS) algorithm. Convolutional neural network for earthquake detection and location (ConvNet). Seismic wave event detection algorithm (SWEDA). Software algorithms, automated information system. Compressive sensing (CS), reconstruction of seismic data challenges.	
**Methods used in experimental analysis**	**Mechanism**	EEW Bayesian approach. Efficient algorithm to reliably detect and locate earthquakes. CS challenges: (1) developing sparse realistic models for seismic data considering the noise recorded in seismic stations; (2) accurately implementing non-uniform sampling, but the acquisition geometry and time basis for both sources and receivers has to be known, as well as other aspects related to the signal-to-noise ratio (S/N).
	**Protocols**	Seedlink protocol adopted for data transmission. Data exchange protocols: SeisComP, SEEDlink, Earthworm package SW InterNAQS nanometrics.
	**Formats**	Metadata formats: XML, CSV, and XSL. Waveforms of seismic vibrations: mini-SEED formats and SAC. Calibration data of recorders: SEED formats, RESP, and PZ. Catalogs of recorded seismic events: QuakeML formats.
	**Topologies**	Embedded suite of SeisComp3 modules. Software application for Reftek monitoring network and RTPD utilities. Accelerometer mesh for seismic prediction in rise buildings. Training sets compared with earthquake catalogs. Simulation of EEW models using recurrent neural netwoks.
**Application Area (Scope)**	Monitoring in real time by seismological organizations, based on the SeisComp3 system. The ConvNetQuake neural network achieves probabilistic event detection and location using a single signal. Good performance for earthquake detection and location depends on the size of the training set. (CS) applied in sparse structure signal sparsity, randomized sampling, optimization-based signal recovery, and perspectives on applications to seismic data acquisition and processing, in contrast to the Shannon–Nyquist sampling theorem.	

## Data Availability

Not applicable.

## Abbreviations

The following abbreviations are used in this manuscript:

SDC	seismological data center
EEW	earthquake early warning
WSN	wireless sensor network
SEED	Standard for the Exchange of Earthquake Data
MSEED	SEED including raw waveform data
SEG-Y	Society of Exploration Geophysicists format
RTPD	REFTEK Protocol (RTP) server
JSON	JavaScript Object Notation
XML	Extensible Markup Language
USGS	United States Geological Survey
IRIS	Incorporated Research Institutions for Seismology
IASPEI	International Association of Seismology and Physics of the Earth’s Interior
CTBTO	Comprehensive Nuclear Test Ban Treaty Organization
FDSN	International Federation of Digital Seismograph Networks
IG-EPN	Instituto Geofísico, Escuela Politécnica Nacional, Ecuador
SGC	Servicio Geológico Colombiano
IGP	Instituto Geofísico de Perú
CSN	Centro Sismológico Nacional, Chile
